# Application of artificial neural networks for enhancing *Aspergillus flavipes* lipase synthesis for green biodiesel production

**DOI:** 10.1016/j.heliyon.2023.e20063

**Published:** 2023-09-19

**Authors:** Mohammad M. El-Metwally, Gamal M. Abdel-Fattah, Fatimah O. Al-Otibi, Dina K.H.EL. Khatieb, Yosra A. Helmy, Youssef M.M. Mohammed, WesamEldin I.A. Saber

**Affiliations:** a Botany and Microbiology Department, Faculty of Science, Damanhour University, Damanhour, 22511, Egypt; b Botany Department, Faculty of Science, Mansoura University, Mansoura, Egypt; c Botany and Microbiology Department, Faculty of Science, King Saud University, Riyadh, 11451, Saudi Arabia; d Department of Veterinary Science, Martin-Gatton College of Agriculture, Food, and Environment, University of Kentucky, Lexington, 40546, Kentucky, USA; e Microbial Activity Unit, Microbiology Department, Soils, Water and Environment Research Institute, Agricultural Research Center, Giza, 12619, Egypt

**Keywords:** Lipolytic activity, Response surface methodology, Fungi, Optimization, Modeling, Box-behnken design

## Abstract

Biodiesel is a sustainable, and renewable alternative to fossil fuels that can be produced from various biological sources with the aid of lipases. This study developed a simple and novel fungal system for lipase biosynthesis to be used for catalyzing the oily residuals into biodiesel, employing the artificial neural network (ANN), and semi-solid-state fermentation (SSSF). *Nigella sativa* was selected among agro-industrial oily residuals as a substrate for lipase biosynthesis by *Aspergillus flavipes* MH47297. The effect of cultural humidity (X1), the surfactant; Brij 35 (X2), and inoculum density (X3) on lipase biosynthesis were researched based on the matrix of Box-Behnken design (BBD). The ANN together with a new fungal candidate and SSSF were then applied for the first time to model the biosynthesis process of lipase. The optimum predicted cultural conditions varied according to the model. The optimum predicted conditions were estimated separately by BBD (X1 = 5.8 ml water/g, X2 = 46.6 μl/g, and X3 = 62156610 spore/g) and ANN (X1 = 5.4 ml water/g, X2 = 54.2 μl/g, and X3 = 100000000 spore/g) models. Based on the modeling process, the response of lipase was calculated to be 214.95 (BBD) and 217.72 U (ANN), which revealed high consistency with the experimental lipase yield (209.13 ± 3.27 U for BBD, and 218 ± 2.01 U for ANN). Despite both models showing high accuracy, ANN was more accurate and surpassed the BBD model. Gas chromatography analysis showed that lipase successfully converted corn oil to biodiesel (29.5 mg/l).

## Highlights

• Pioneer *Aspergillus flavipes* for lipase biosynthesis
• Semi-solid-state fermentation for lipase biosynthesis
• Box-Behnken design for optimizing fungal lipase biosynthesis.
• First report on using artificial neural networks for modeling lipase biosynthesis.
• Biodiesel production by *Aspergillus flavipes* lipase.

## 1 Introduction

Fungi are fundamental influences in our lives, and one of their most important contributions is the production of enzymes. Among many fungal enzymes, lipase has received significant attention with various industrial applications, including detergent, food, flavor, pharmaceuticals, cosmetics, agrochemicals, biosensors, bioremediation, and biodiesel [1,2].

Biodiesel is one of the promising renewable bioenergy, and lipase enzymes are one of the fundamental ingredients in biodiesel production from oily wastes. Biochemically, lipase (triacylglycerol acylhydrolases, E.C. 3.1.1.3) catalyzes triacylglycerol into fatty acids and glycerol, the process initiates the conversion of oils and fats for biodiesel production [3]. However, the biosynthesis of microbial lipase can vary depending on the cultivation conditions, medium composition, and specific strain [4].

Biodiesel, fatty acid alkyl ester, fatty acid ethyl ester, or fatty acid methyl ester, is created from a decomposition process known as alcoholysis (methanolysis or ethanolysis) of triacylglycerol with methanol or ethanol alcohol [5]. Biodiesel can be produced from waste and non-edible vegetable oil to decrease the expenses of production. This practice helps prevent conflicts between energy, and food security, as well as, serves as an important issue to reduce pollution by recycling oil wastes [6,7]. Biodiesel represents about 5% of the world's biofuel manufacture, with a share of around 20% of the total biofuel production [8]. The major challenge associated with biodiesel synthesis is the higher cost of fabrication, compared to fossil diesel, thus, it is essential to reduce the production cost, improve the production technology, reduce capital cost, and use an efficient catalyst [6].

Optimization of the fermentation medium is a vital step to improve enzyme yield and reduce the overall cost as well. Solid-state fermentation utilizes renewable resources like agricultural and food waste for higher productivity and product stability. However, it faces challenges such as low mass-transfer efficacy and substrate inhibition due to high sugar content. The semi-solid-state fermentation (SSSF) technique addresses these issues by increasing the content of water, leading to improved nutrient availability and fermentation control. SSSF offers high productivity, transformation efficiency, simplicity, and minimal pollution compared to traditional solid-state fermentation methods [9].

Most of the previous work on the optimization for lipase production is based on the traditional design of one-variable at a time (OVAT), which is an approach that successively studies only a single independent variable at one time on the response variable. Therefore, OVAT is time-consuming and limited in providing a comprehensive understanding of the process interaction of the studied system [10].

On the other hand, response surface methodology (RSM) is a design procedure used to model the relationship between multiple inputs and the response or output of a system. It is frequently utilized to enhance system performance by finding the best settings for a set of influential variables [11]. RSM provides an efficient statistical means for the design and analysis of trials to optimize the process performance. Thus, allows insight into a strong spot where the specification parameters can be maximized while minimizing the operation parameters [10,12].

Two major designs of RSM are known (the central composite designs (CCD), and BBD)) to fit full quadratic models. The BBD is a statistical experimental design technique that is widely used in research and development, particularly in the field of optimization. It allows researchers to investigate the main effects and interactions of multiple factors simultaneously. BBD is an important tool in experimental design and optimization, providing a balance between efficiency flexibility, and accuracy. Contrarily to CCD, BBD does not include axial points in the design space; so, BBD has fewer design trials thus reducing the cost and time required for experimentation, than CCD, enabling resource efficiency and fewer experiments needed [13,14]. In BBD, the axial points (extreme runs) are not necessary because the studied response region is known and well-behaved. As a result, extreme tests are avoided, and factors excel within tested limits. BBD is not sensitive to the order of the experimentation (rotatable nature), which makes it safe, and more robust to noise, and does not require running the design in a specific order [15,16].

Artificial neural network (ANN) stands as a central component and a primary key tool employed within the realm of artificial intelligence. ANN could be considered a developed variant of RSM. Similar to the human brain, an ANN can intricately analyze and process data, constructing effective computational models through fully connected nodes within the layers. This approach facilitates the learning of data patterns, leading to precise decision-making based on investigative data [17]. During the construction of an ANN model, the initial step involves choosing the network architecture, followed by establishing hidden layer(s) with an appropriate number of neurons. Subsequently, the network undergoes a training process to learn and comprehend the data patterns. After successful training, the resultant ANN model is subjected to validation and verification before gaining approval as a predictive model [13,18]. The learning process of ANN relies on identifying various patterns within the data and discerning variations to establish which pattern successfully reaches the intended goal. This procedure is controlled by an intelligent backpropagation method, leading to the creation of the desired output model for goal achievement. This modeling approach is characterized by enhanced accuracy and the potential to effectively substitute other modeling systems [12,14,19,20].

Generally, no previous work on the SSSF of agro-industrial oily residuals for the production of fungal lipase using ANN. This study presents a groundbreaking approach for optimizing the biosynthesis of fungal lipase for use in the production of biodiesel. In this study, the agro-industrial oily residual of *Nigella sativa* was selected as a substrate for lipase biosynthesis by a new lipolytic *Aspergillus flavipes* MH472977 under SSSF. The process involves a unique system developed through SSSF combined with an innovative modeling approach of ANN technology. The study extended to the application of the resulting fungal lipase in converting corn oil to biodiesel.

## 2 Materials and methods

### 2.1 Fungal isolate

The fungus was isolated from agro-industrial oil residuals as a lipolytic fungus in a prior study [21] and identified morphologically as *Aspergillus flavipes*. The molecular sequence of the fungus was deposited in the NCBI GenBank under the accession number MH472977. *Aspergillus flavipes* was maintained in a PDA medium (Difco) under refrigeration at a temperature of 4 °C. Every two months, the fungus was subcultured to keep its viability.

### 2.2 The agro-industrial residuals

As cheap and economical substrates, seven agro-industrial oily residuals (*Eruca sativa, Nigella sativa, Sesamum indicum, Glycine max, Gossypium barbadense, Linum usitatissimum*, and *Olea europaea*) were individually screened as a solid substrate for lipase production by *A*. *flavipes*. The residuals were obtained after oil extraction from “El-Nasr Company for Natural Oil” in Damanhur, El-Behira Governorate, Egypt. The residuals of the oils served individually as a substrate in a screening trial to select the most appropriate for lipase production by *A*. *flavipes*.

### 2.3 Fermentation procedure for lipase production

The SSSF was applied in the current study for lipase production as an innovative bioprocessing approach that garnered considerable attention in recent years. SSSF is a type of fermentation process that lies between solid-state fermentation and submerged or liquid-state fermentation. Unless otherwise stated, the core medium used in the screening experiment was composed of 1 g of each agro-industrial residue type moistened with 6 ml of tap water in 250-ml-Erlenmeyer flasks and autoclaved (121 °C, 20 min). Inoculation was carried out using fungal spore suspension (10^6^ spore/gram substrate), and incubation at 28 °C for 4 days. The produced enzyme was eluted by adding 20 ml water to each ﬂask and rotated at the incubator shaker (200 rpm) for 1 h at room temperature. Afterward, the suspension was filtered using a double-layer muslin cloth and subsequently subjected to centrifugation (5000 rpm, 20 min). The resulting filtrate, containing the extracellular lipase was preserved at −20 °C for further study.

### 2.4 Assay of lipase

Lipolytic activity was determined using 4-nitrophenyl palmitate [22]. In brief, a mixture of 1.8 ml of 0.1 M Tris-HCL buffer (pH 8), 0.15 M NaCl, and 0.5% Triton X-100 was prepared and pre-incubated with 100 μl of crude lipase at 37 °C. Subsequently, 20 μl of 50 μM 4-nitrophenyl palmitate, dissolved in acetonitrile, was added to the reaction mixture as a substrate and incubated at 37 °C for 30 min. The liberated *p*-nitrophenol (*p*-Np) was quantified at 410 nm. One lipase unit (U) was determined as the lipase amount that liberates 1 mM of *p*-Np per minute per gram of dry substrate under the assay situations.

### 2.5 Design of Box-Behnken matrix

The experimental matrix of the BBD was constructed to maximize the biosynthesis of lipase by *A*. *flavipes* grown on the selected oil residue (*Nigella sativa*) based on the previous screening trial. The experiment runs were experimentally performed, and the lipase activity was assayed. Three variables, including humidity (amount of water added to gram substrate), surfactant (Brij 35) concentration, and inoculum density, were chosen for the optimization of the fermentation conditions for maximization of lipase production, applying the matrix of BBD. The objective was to understand the relationships between the three independent variables and lipase synthesis thus the optimum cultural conditions for highest lipase biosynthesis by the selected *A. flavipes* could be determined.

The three factors were examined at low (−), middle (0), or high (+) levels for each factor. The runs of the experimental matrix, which includes coded and uncoded levels, represent the different combinations of levels for each independent variable are shown in Table (1). Each trial was carried out in triplicates. Once performing the experiments, lipase production was assayed in response to the three-factor matrix.

#### 2.5.1 Modeling lipase production using BBD

To define the significant factors and the goodness of the model, the analysis of variance (ANOVA) and determination coefficient (R^2^) were calculated. The gathered data were statistically analyzed to elucidate the association between the three tested variables and lipase biosynthesis, as well as the prediction of the optimum level of each variable. The function of the second-order polynomial quadratic model (Equation (1)) was applied:

(1) $${{Y=\beta }_{0}+\sum \beta }_{i}X_{i}+\sum \beta_{ij}X_{i}X_{j}+\beta_{ii}X_{i}^{2}$$

where *Ү* is the predicted lipase, *β*_*0*_ is the model constant, *β*_*i*_ is the linear coefficient, *β*_*ij*_ is the cross-product coefficient, *β*_*ii*_ is the quadratic coefficient, and *X*_*i*_, and *X*_*j*_ are the tested variables.

#### 2.5.2 Modeling lipase biosynthesis using ANN

Generally, machine learning requires historical data for the modeling process. The BBD matrix data was utilized for machine learning purposes and to develop an artificial intelligence-based predictive model. To create the prediction model, the experimental dataset obtained from the BBD matrix was used to train a fully connected ANN with a single hidden layer. The nodes have the activation function of hyperbolic tangent sigmoid; exp(-x^2^). The forecast model was a fully connected multilayer perceptron algorithm. The results of BBD were divided into three datasets. The first was utilized for training to reduce the error and establish weights at each neuron. The second was used for validation purposes to halt the ANN training and select the best model. Another third dataset was designated as an outer dataset that was used to examine the ANN strength and serve as the final evaluation of forecast capabilities. The model was not trained using this external dataset.

The ANN architecture was designated to contain three layers as 3-h-1. Three neurons (cultural humidity, the surfactant; Brij 35, and the inoculum density), represent the input layer. The response layer is composed of one neuron (lipase biosynthesis by the fungus). An additional hidden layer was created between the two existing layers and evaluated by testing with a range of neurons spanning from 3 to 10.

Utilizing the trial-and-error system for ANN training, various ANN-specific parameters i.e., learning rate, and method, and the ideal neuron number in the hidden layer, were examined at 100 tours for each training phase. Accordingly, the ideal structure for the best model was determined. The trial-and-error was utilized to train the ANN until the lowest error values were achieved for both the root mean square error (RMSE) and mean absolute deviation (MAD), accompanied by the highest value of R-squared. The performance of the trained network was evaluated by assessing its precision in predicting outputs that either like or closely resemble the actual value of lipase biosynthesis.

#### 2.5.3 Experimental validation

The best values of the tested variables that maximize lipase biosynthesis were estimated by the fitted BBD and ANN models, and then the expected lipase yield was inferred for each model. The validation trials were performed, applying the optimum settings for each predicted model in the laboratory in triplicates. The experimental results of both models were compared with the predicted lipase to test the precision of both models.

### 2.6 Biodiesel production by onsite lipase

The fungal lipase obtained based on the previous optimum conditions - inferred by the ANN model - was incorporated in the lipase biosynthesis trial. The reaction was carried out according to Panichikkal et al. [23] with some modifications. A mixture of corn-oil, and methanol at a molar ratio of 1: 9 was added to a 250-ml conical flask. The mixture was incubated with the prepared fungal lipase as a biocatalyst at a fixed proportion of 1000 U under constant shaking (200 rpm) at 45 °C for a maximum of 24 h. Lipase functions by breaking down the ester bond of triglycerides, which results in the synthesis of fatty acid methyl esters. The mixture was subsequently transferred to a separating funnel, where it underwent gravity separation and was separated into two layers. The top layer, which was the biodiesel layer, separated from the bottom layer of glycerol to remove any extra methanol, and the biodiesel layer was gathered. This layer was then washed three times with warm, deionized water to eliminate any dissolved glycerol and impurities. The washed aliquots were subsequently dried over anhydrous sodium sulfate and placed in an oven at 110 °C for 20 min to ensure complete drying. Finally, the pure and dried biodiesel was stored in a controlled room temperature environment (below 28 °C) for future analysis. The resulting biodiesel aliquots were analyzed using gas chromatography (Shimadzu, GC 17-A, Shimadzu Corporation, Tokyo, Japan) at the National Institute of Oceanography and Fisheries, Alexandria, Egypt.

### 2.7 Software and mathematical analysis

Experiments of fungal lipase biosynthesis were performed in triplicates and the data were presented as the average ± standard deviation. The matrix and the statistical analysis of the BBD data were established using the software; JMP® Pro 17 (JMP®, SAS Institute Inc., Cary, NC). In addition, the same data were used to train the ANN, conduct machine learning procedures, build the ANN topology, and implement the training, validation, and testing protocols, as well as, calculate the optimum predicted conditions.

## 3 Results

### 3.1 Lipolytic activity of *A. flavipes* on oily residuals

**Fig. 1:**
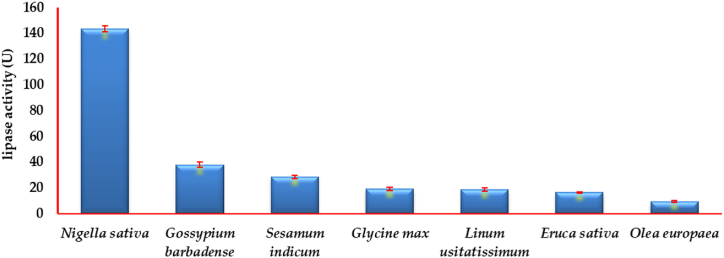
Production of extracellular lipase on semi-solid-state fermentation by *Aspergillus flavips* in different agro-industrial oily residuals at 28 °C for 5 days.

Under SSSF, seven oily agro-industrial residuals were screened as fermentation substrates for lipase biosynthesis (Fig. 1), *Nigella sativa* is the most suitable substrate to produce lipase followed by *Gossypium barbadense* and *Sesamum indicum* with lipase activity 143.4, 38.0, and 28.67 U, respectively. Thus, the *Nigella sativa* residual was selected in the next optimization trials using BBD.

### 3.2 Modeling lipase biosynthesis by BBD

The interaction effect of three cultural conditions (cultural humidity (amount of water added to gram substrate), Brij 35 concentration, and inoculum density) on lipase biosynthesis by *A. flavipes* was investigated. *Nigella sativa* was chosen as a fermentation substrate. The BBD experimental design was applied to three independent variables, each at three different levels. Table (1) displays the design (15 runs) of the coded, and true variables as well as the results of lipase biosynthesis. Data displayed a substantial disparity in lipase production, depending on the levels’ combination of the three independent variables The production ranged from 81.99 U (run 2) to 218.51 U (run 13).

#### 3.2.1 Parameters contribution

The obtained BBD data were analyzed to find out the contribution of each of the input parameters (single, interaction, and quadratic) in the lipase production (Table 2). The single parameters showed that the surfactant (X2) was the most important factor followed by inoculum density (X3), and cultural humidity (X1), the latter showed no contribution to the single factors’ effect. The interaction effect of X1*X2 had the highest contribution to lipase biosynthesis. Finally, the quadratic effect of X2*X2 was the most effective parameter, followed by X1*X1, and X3*X3.

#### 3.2.2 ANOVA and regression analysis

Data from Table (2) of ANOVA show that the BBD model is significant (*F-*value = 52.130 with a low *P-*value of 0.0002), on the other hand, the lack of fit error is, statistically, insignificant (*P*-value = 0.312). The model terms, R^2^ and adjusted-R^2^, were found to be very high (0.9895, and 0.9705, respectively).

All the model terms were significant except for cultural humidity, inoculum density, and their interaction. Accordingly, the regression coefficients of the various model terms were estimated and fitted to a polynomial function. The predicted lipase biosynthesis by *A. flavipes* can be computed by Equation (2) based on the second-order regression model (Equation (1)) (coded values):

(2) $$Y=212.82+0.15\left(X1\right)‒13.09\left(X2\right)+2.12\left(X3\right)+45.34\left(X1X2\right)‒3.94\left(X1X3\right)‒17.74\left(X2X3\right)‒31.60{\left(X1\right)}^{2}‒47.27{\left(X2\right)}^{2}‒16.74{\left(X3\right)}^{2}$$

where Y is the predicted lipase, X1; cultural humidity (ml water/g), X2; the surfactant Brij 35 (μl/g), X3; inoculum density (spore/g).

It is obvious that the model terms; X2, X1X3, X2X3, and all quadratic terms, had negative coefficients, whereas the other terms (X1, X3, and X1X2) had positive coefficients. However, Equation (2) was used for the generation of the predicted levels of lipase, which were extremely close to the investigational values and therefore lower residuals (Table 1).

**Table 1:** Three-factor matrix used for lipase biosynthesis using semi-solid-state fermentation by *Aspergillus flavipes* on *Nigella sativa* residuals and the predicted value of every point of Box-Behnken and ANN models.

Run^a^		Independent variable (Coded value)			Lipase (U)				
					Actual ± SD	Box-Behnken		ANN	
		X1	X2	X3		Predicted	Residual	Predicted	Residual
1	Training	−1	−1	0	192.43 ± 2.62	192.22	0.21	192.96	−0.53
2	Validation	−1	1	0	81.99 ± 1.95	75.37	6.62	79.31	2.68
3	Training	1	−1	0	95.23 ± 2.44	101.85	−6.61	94.51	0.73
4	Validation	1	1	0	166.13 ± 3.18	166.34	−0.22	157.24	8.88
5	Training	0	−1	−1	146.46 ± 2.06	142.03	4.43	145.69	0.77
6	Validation	0	−1	1	183.73 ± 5.00	181.76	1.97	181.58	2.15
7	Validation	0	1	−1	149.37 ± 3.41	151.34	−1.97	150.21	−0.84
8	Training	0	1	1	115.67 ± 2.75	120.10	−4.43	115.58	0.09
9	Training	−1	0	−1	153.62 ± 3.93	158.27	−4.65	153.70	−0.08
10	Training	1	0	−1	168.63 ± 4.30	166.45	2.19	171.01	−2.38
11	Training	−1	0	1	168.21 ± 3.68	170.39	−2.18	168.25	−0.04
12	Training	1	0	1	167.46 ± 3.27	162.81	4.64	168.13	−0.68
13	Training	0	0	0	218.51 ± 4.00	212.82	5.69	212.43	6.08
14	Validation	0	0	0	211.46 ± 2.36	212.82	−1.36	212.43	−0.98
15	Training	0	0	0	208.48 ± 5.55	212.82	−4.34	212.43	−3.96
Level		Actual value			X1; Cultural humidity (ml water per gram substrate) X2; Surfactant concentration; Brij 35 (μl/g) X3; Inoculum density (spore/g)				
Low (−1)		5	40	10000					
Middle (0)		6	60	50005000					
High (1)		7	80	100000000					

a Ten experimental runs were used for ANN training, while 5 experimental runs were served for the ANN validation.

**Table 2:** The contribution, and ANOVA of data of Box-Behnken matrix for lipase production on *Nigella sativa* by *A*. *flavipes* under semi-solid state fermentation conditions.

Source		Contribution, %	Degree of freedom	Sum of squares	Mean square	F-ratio	*P-*value
Overall model		98.95	9.00	22564.24	2507.14	52.13	0.00
Error	Lack of Fit	0.82	3.00	187.41	62.47	2.36	0.312*
	Pure	0.23	2.00	53.06	26.53		
Single	X1	0.00	1.00	0.18	0.18	0.00	0.9536*
	X2	6.01	1.00	1370.00	1370.00	28.49	0.00
	X3	0.16	1.00	36.08	36.08	0.75	0.426*
Interaction	X1*X2	36.05	1.00	8221.05	8221.05	170.94	<.0001
	X1*X3	0.27	1.00	62.09	62.09	1.29	0.3074*
	X2*X3	5.52	1.00	1259.19	1259.19	26.18	0.00
Quadratic	X1*X1	11.96	1.00	3686.89	3686.89	76.66	0.00
	X2*X2	34.44	1.00	8251.01	8251.01	171.56	<.0001
	X3*X3	4.54	1.00	1034.33	1034.33	21.51	0.01
Model evaluation statistics							
Determination coefficient (R^2^)				0.9895			
Adjusted-R^2^				0.9705			

X1; cultural humidity (ml water/g), X2; the surfactant; Brij 35 (μl/g), X3; inoculum density (spore/g).

### 3.3 Modeling lipase production by ANN

The innovative advanced modeling procedure based on artificial intelligence was used to model the BBD data for maximizing *A. flavipes* lipase production. For this purpose, a fully connected multilayer feed-forward ANN was used to construct a predictive model within the neural network platform.

For best ANN construction, the machine learning was performed with a trial-and-error procedure, and after several trials (each of 100 tours). The squared learning method at 0.1, with three layers, was the best combination of the ANN topology with the best holdback propagation at 0.3333, which divided the data, the first set (10 training runs) to reduce the prediction error, and created neural weights. The second set (5 validation runs) is to stop ANN training and select the supreme model.

**Fig. 2:**
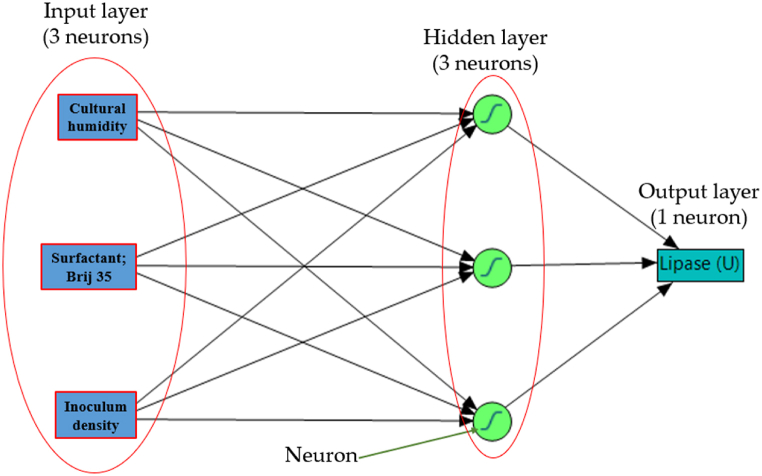
The layout ANN of lipase production by *Aspergillus flavipes*, contains an input (3 nodes), a hidden (3 nodes), and an output (one node) layers. Cultural humidity (ml water/g), the surfactant; Brij 35 (μl/g), inoculum density (spore/g).

In addition, the activation function of the hidden layer employs a hyperbolic tangent sigmoid that is shared by all nodes. Thus, the topology of the three-layer ANN denoted as 3-3-1 (Fig. 2), was found to be the optimal topology. The input layer consists of three neurons, which correspond to the number of independent factors that were examined: cultural humidity, Brij 35 concentration, and inoculum density. The single neuron in the output layer represents lipase biosynthesis. The single hidden layer confirmed superior performance when using the activation function of hyperbolic tangent sigmoid on the three hidden neurons.

To assess the model's generalizability, the ANN was trained until the R^2^ value was the highest, achieving 0.9933. The trained network's ability to accurately predict outputs that were either like or at least so close to the lipase value was evaluated. The predicted values were computed using the ANN and presented in Table (2). The values predicted by ANN displayed high agreement with the investigational ones with lower residual points than those calculated by the BBD model.

### 3.4 Evaluating BBD and ANN models

#### 3.4.1 Linear relationships

**Fig. 3:**
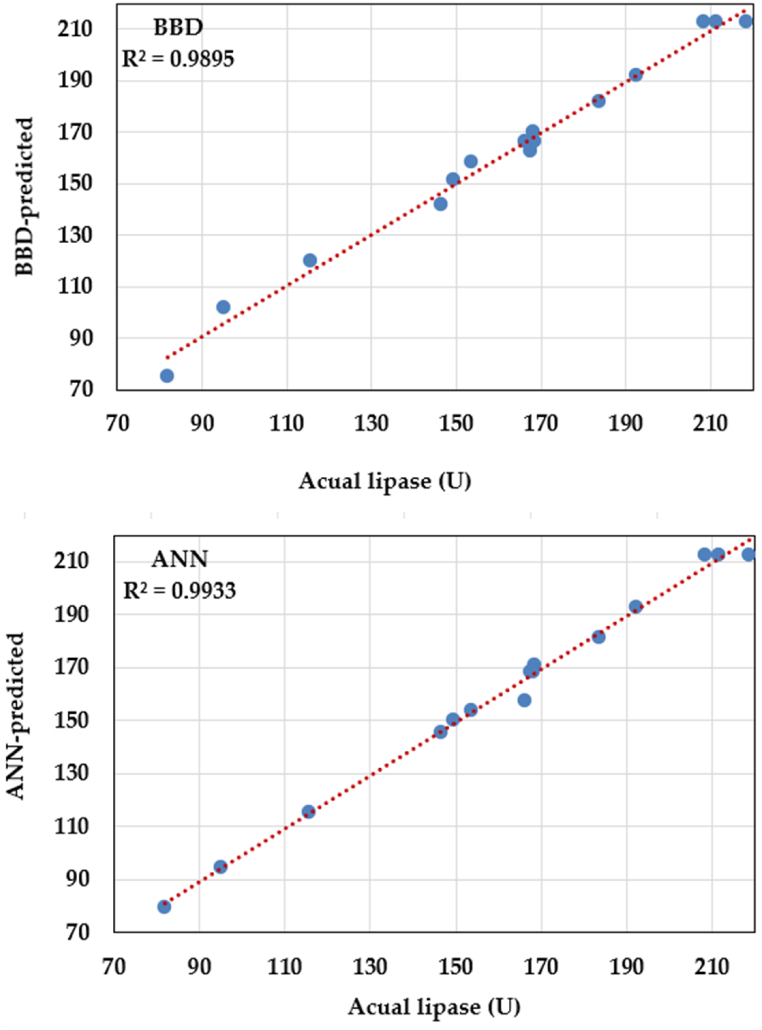
BBD and ANN predicted versus actual values of lipase biosynthesis by *Aspergillus flavipes*.

To assess the fitness of both the both models, their adequacy was compared. The predicted points were plotted against the experimental points (Fig. 3). Both models displayed precise predictions. Points from both models closely align with the perfect prediction line, suggesting the model faithfully approximates actual experimental data. Nevertheless, linear regression demonstrated that the ANN model's forecasts are notably closer to the prediction line in comparison to the BBD model.

#### 3.4.2 Residual analysis

**Fig. 4:**
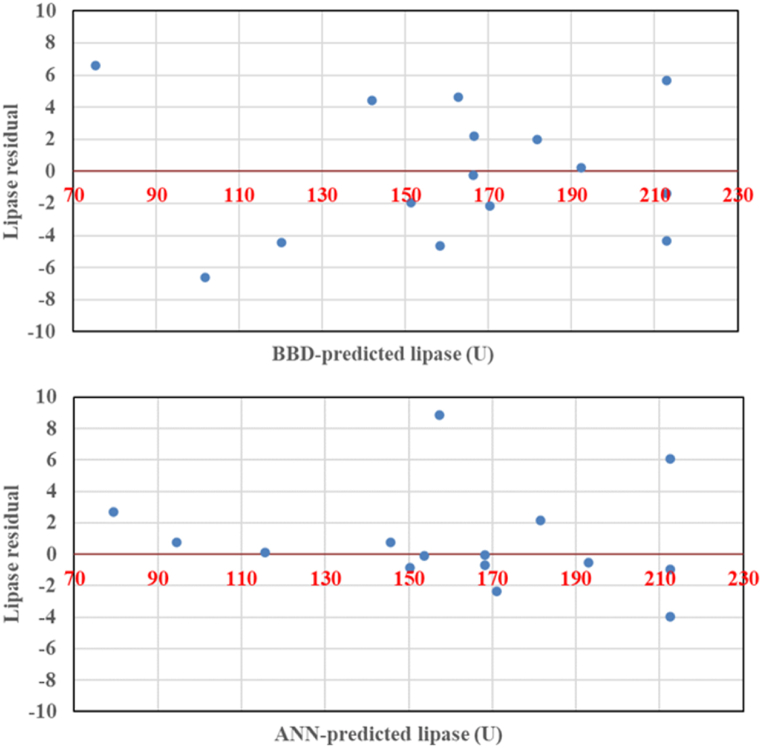
Plots of the BBD and ANN predicted versus residual values of fungal lipase biosynthesis of the experimental data.

The residual test was accomplished for the assessment of the adequacy and fitness of both models to predict lipase biosynthesis by the selected fungus. The residuals versus predicted values were drawn. The constructed residual plots (Fig. 4) unveiled an equal spreading of the residuals, and residual data were equally distributed around the 0-axis without linearity.

### 3.5 Mutual relationships

**Fig. 5:**
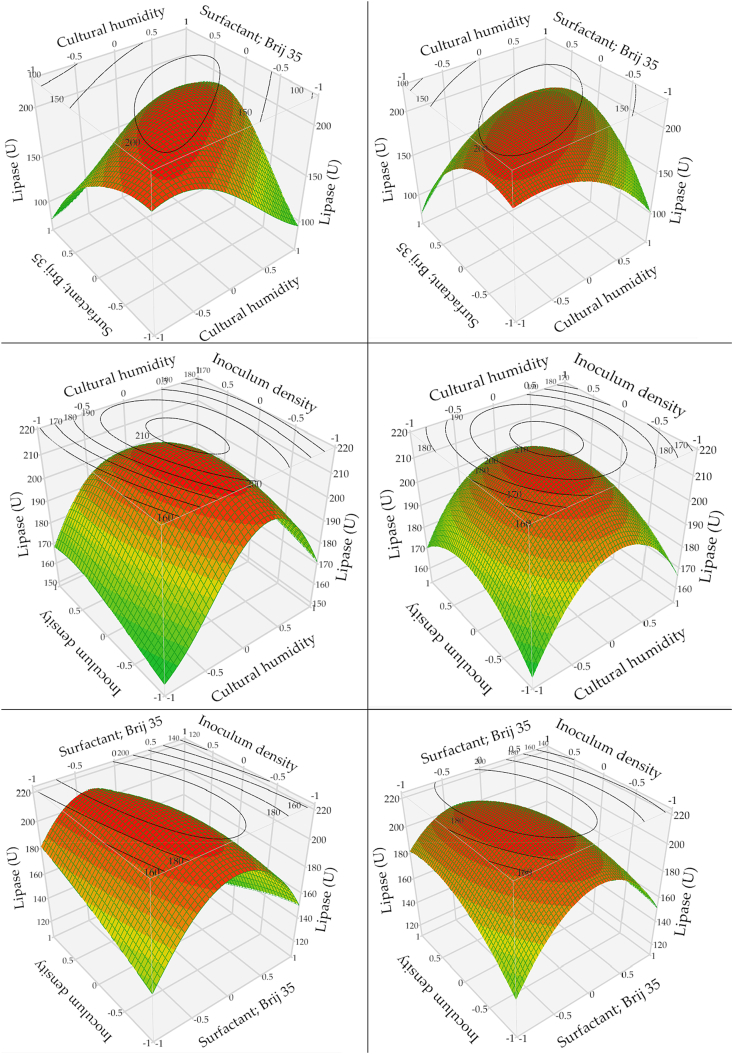
Response surface 3D plots of fungal lipase production, represented as a pairwise pattern of the tested variables, keeping the third-variable constant at its center level, based on the models of ANN (left column), and BBD (right column). Cultural humidity (ml water/g), the surfactant; Brij 35 (μl/g), inoculum density (spore/g).

To understand the mutual effects between every two tested variables, an analysis of the three-dimensional (3D) graph of BBD and ANN was performed (Fig. 5). Presenting the results in the form of a surface plot helped to imagine the interactive effects of the examined variables. The 3D plots depicted the associations between the variables, signifying an enhancement in lipase biosynthesis as the concentration of each variable approaches the optimum level and then declines. The high level of surfactant and humidity has a negative impact on lipase. BBD displays elliptical response curves when comparing each pair of tested factors on the surface plot. Furthermore, lipase biosynthesis reached its highest value around the midpoints of the design. On the other side, 3D plots of ANN showed diverse patterns, and no exact prototype could be caught for every two of the tested variables.

### 3.6 Models’ comparison

Both BBD and ANN models demonstrated robust predictive capability with minimal residuals, indicating their accurate fit to experimental data and reliable predictive performance. To assess the accuracy of the models in predicting fungal lipase biosynthesis, several performance statistical parameters were evaluated during both the training and validation stages, as well as for the overall models (Table 3). It was evident that the ANN model exhibited greater confidence compared to the BBD model, where the overall R^2^ for the ANN model surpassed that of the BBD model. Conversely, the ANN model displayed lower RMSE and MAD values compared to the BBD model.

**Table 3:** BBD and ANN performance statistics, prediction, and actual lipase biosynthesis by *Aspergillus flavipes*.

Model		R^2^	RMSE	MAD	Frequency
Training	BBD	0.9895	4.325	3.939	10
	ANN	0.9955	2.451	1.531	10
Validation	BBD	0.9943	3.269	2.425	5
	ANN	0.9902	4.300	3.107	5
Overall	BBD	0.9895	4.004	3.434	15
	ANN	0.9933	3.189	2.057	15
Predicted optimum conditions					
Factor				Predicted value	
				BBD	ANN
Cultural humidity (ml water/g)				5.8	5.4
Surfactant; Brij 35 (μl/g)				54.2	46.6
Inoculum density (spore/g)				62156610	100000000
Validation of response					
Predicted lipase (U)				214.95	217.72
Experimental lipase (U)				209.13 ± 3.27	218 ± 2.01
Desirability function				0.9470	0.9662

RMSE is the root squared error of the mean. MAD is the absolute deviation of the mean.

### 3.7 Model validation

A verification experiment was conducted to evaluate the modeling process employed in the optimization of lipase biosynthesis, in which the optimal situation was predicted independently by both BBD and ANN models. Subsequently, the calculated values of the three tested factors were verified through experimentation. This validation was conducted under the estimated optimum conditions for the three variables, as outlined in Table (3). The response of lipase was calculated to be 214.95 and 217.72 U, with a desirability value of 0.9470 and 0.9662, for BBD and ANN, respectively. The validation test revealed high reliability with the actual lipase yield, being 209.13 ± 3.27 and 218 ± 2.01 U. The two models exhibited high performance, but ANN exceeded the BBD model.

### 3.8 Production of biodiesel by fungal lipase

**Fig. 6:**
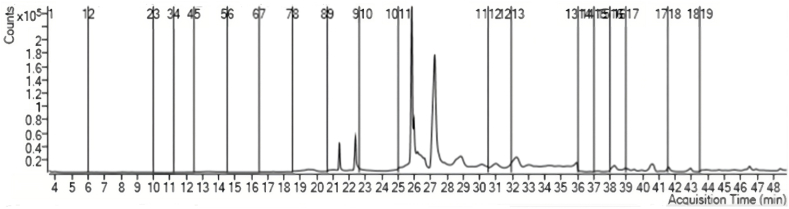
The gas chromatography spectrum of biodiesel produced from the transesterification reaction catalyzed by *A. flavipes* lipase.

Gas chromatography analysis was utilized to explore the level of bioconversion of the corn oil used in biodiesel (Table 4, and Fig. 6). The profile of kinds and concentration, and percent of fatty acid methyl esters, as biodiesel synthesized from the transesterification reaction showed that oleic, lignoceric, caprylic, and behenic acids were the most dominant, being 10.23, 8.77, 7.65, and 7.01%, respectively.

**Table 4:** Profile of fatty acid methyl esters of the biodiesel synthesized from the transesterification reaction of corn oil by *A. flavipes* lipase.

Fatty acid	Number of carbon atoms	Retention time (min)	Concentration (mg/l)	Percent (%)
Caprylic acid	8	6.67	2.26	7.65
Capric acid	10	11.19	0.29	0.99
Undecanoic acid	11	12.06	0.22	0.75
Lauric acid	12	14.00	0.20	0.67
Tridecanoic acid	13	15.59	0.27	0.92
Myristic acid	14	17.35	0.40	1.36
*cis*-10-Pentadecenoic acid	15	19.50	1.46	4.93
Pentadecanoic acid	15	19.53	0.44	1.49
Palmitoleic acid	16	20.98	0.78	2.65
Palmitic acid	16	21.39	0.09	0.31
*cis*-10-Heptadecenoic acid	17	22.65	0.89	3.03
Heptadecanoic acid	17	23.88	0.59	2.01
Gama-Linolenic acid	18	25.85	1.67	5.66
Linolenic acid	18	25.85	1.61	5.44
Oleic acid	18	25.97	3.02	10.23
Elaidic acid	18	25.85	1.03	3.50
Stearic acid	18	26.65	0.98	3.31
*cis*-5,8,11,14,17 Eicosapentaenoic acid	20	31.00	1.73	5.85
Arachidic acid	20	33.04	0.95	3.20
Heneicosanoic acid	21	36.97	1.09	3.70
*cis*-4,7,10,13,16,19 Docosahexaenoic acid	22	37.26	1.90	6.45
Behenic acid	22	39.51	2.07	7.01
Tricosanoic acid	23	41.75	1.29	4.37
Nervonic acid	24	43.89	1.69	5.74
Lignoceric acid	24	44.47	2.59	8.77
Summation			29.50	100

## 4 Discussion

Recently, microbial lipases have attracted attention due to the fast advancement in enzyme technology. Lipase, which belongs to a group of enzymes that catalyze the hydrolysis triglycerides, has enormous industrial potential and can be utilized across various sectors, such as the medical, pharmaceutical, cosmetic, chemical, leather, and paper manufacturing industries, and biosurfactant [[1], [2], [3]].

Biodiesel offers numerous environmental benefits as an energy solution that involves using vegetable oils as engine fuels in place of petro-diesel [24]. The main challenges in the production of biodiesel are linked to the primary raw materials and catalysts employed in the process [25]. The major biological catalysis is lipases, however, filamentous fungi and yeasts produce 50% of commercial lipases [26].

Therefore, the current study reported a novel scenario using a combination of BBD and ANN for the optimization of lipase biosynthesis by *Aspergillus flavipes* grown on *Nigella sativa* as an agro-industrial oil residual. The resulting lipase approved its efficiency as a green catalyst in biodiesel generation. In general, filamentous fungi are preferred over bacterium for lipase production, since fungi can efficiently break down materials in the natural environment into carbon and nitrogen at a greater percentage than bacteria [27].

### 4.1 Fungal fermentation of oil residuals

The implementation of an appropriate bio-fermentation mode can significantly reduce the overall cost of bio-fermentation processes. The traditional method of submerged fermentation has been widely utilized worldwide for several decades due to its ability to maintain various parameters such as temperature, pH, humidity, and airflow, resulting in a highly homogeneous culture medium [9]. However, its extensive application is restricted due to low product concentration and complicated downstream processing. To some extent, solid-state fermentation can overcome these limitations by growing microorganisms on a solid substrate without free water [28].

Practically, solid-state fermentation can utilize renewable and inexpensive resources, such as forestry and agricultural waste, and food waste with higher productivity, product stability, and minimum contamination [28]. But in some cases, low mass-transfer efficiency, and substrate inhibition due to its high sugar content make some disadvantages. As a result, attempts have been made to improve this method by a limited increase of the water content to enhance nutrient availability and fermentation control, leading to the development of the SSSF method. This modified technique offers high both system productivity and transformation efficiency, as well as simple operation, and minimal secondary pollution [9].

Thus, in our study, 7 oily agro-industrial solid residuals were screened using SSSF. The proximate oil content (%) was 35.4 (*Nigella sativa*), 20 (*Eruca Sativa*), 48–55 (*Sesamum indicum*), 20 (*Glycine max*), 27 (*Gossypium Barbadense*), 37 (*Linum usitatissimum*), and 33–39 (*Olea europaea*). *Nigella sativa* yielded maximum lipase biosynthesis by the fungal candidate. Compared with previous results, lower lipase activity was reported by several fungi under various fermentation conditions [27]. In contrast, another work reported maximum lipase production of 1152 U after 96 h of incubation by *Aspergillus* spp [29].

### 4.2 Modeling lipase biosynthesis by BBD

Data from BBD showed that the maximum lipase production occurred at the center point, indicating the accuracy of the factors’ selection as well as, their level range. To assess the BBD matrix model, the data were fitted with a second-order polynomial function using ANOVA. The results of the ANOVA indicated that the quadratic regression model was a good fit, as demonstrated by its significant P-value and lack of significant error in the lack of fit test. Furthermore, the accuracy of the model terms was confirmed, with most of the terms being significant, as evidenced by their high F-values and low P-values (≤0.05). Additionally, the lack of fits was insignificant in comparison, indicating the significance of the model [30].

Commonly, the P-value of ≤0.05 is employed to detect the significance of the coefficients of various model terms, which is necessary for understanding the pattern of single and mutual interactions between the tested variables [13]. The significance level at the P ≤ 0.05 threshold signifies a 5% risk of incorrectly concluding the presence of an association between the tested variables and lipase production when no such association exists [14,19]. The same principle applies to individual variables as well.

### 4.3 Evaluation of the BBD model

The appropriateness of the data is indicated by the insignificant lack of fit. When the model exhibits a high F-value along with a lack of fit that is not statistically significant, it suggests that the model is suitably fitting the data. The null hypothesis (H_0_) of the F-test assumes that there are no differences among the model terms, and the alternative hypothesis (H_1_) is used when there are differences among model terms (rejection of H_0_). It is important to note that the P-value, which is determined by the F-value, is used in all hypothesis tests to decide whether to accept or reject the H_0_. A small P-value (≤0.05) provides strong evidence to reject H_0_, whereas a large P-value (>0.05) suggests accepting H_0_. So, the P-value serves as a guideline for disregarding data that does not meet a specified significance level [14,31,32]. Based on the present data, there is strong evidence to reject H_0_, suggesting a significant effect of the tested factors on lipase production. As a result, the non-significant lack of fit, high adjusted R^2^ value of the model, and high F-value demonstrate the model's accuracy and reliability in predicting lipase production by *A. flavipes*.

Another round of model evaluation was conducted based on the values of R^2^ and adjusted-R^2^, which should both be positive with a value of ≥0.75 and relatively close to each other [20,31]. The high values of R^2^ (0.9895) and adjusted-R^2^ (0.9705) indicate the model fitness that demonstrates a strong agreement between the predicted and experimental values of lipase production. It is important to note that as the values of R^2^ and adjusted R^2^ increase, the model becomes more appropriate and the relationships between the tested variables and the response become more accurate. Thus, the model can explain 97.05% of the variation in lipase response under the current fermentation conditions. However, a high R^2^ value does not forever imply a good regression model. Such an assumption can only be true if the adjusted R^2^ value is also relatively high [19].

The estimated coefficients were calculated to generate the second-order polynomial Equation (2) and used for calculating the predicted values. The latter was found to be very close to the actual values of lipase production, suggesting additional evidence for the accuracy of the model, and the design space. When the coefficient values are negative, it indicates an antagonistic or reverse relationship between the fungal lipase production and the tested variable concentration [20]. On the other hand, positive coefficient values suggest a synergistic relationship between the variable concentration and the biosynthesis of fungal lipase in the tested design space.

### 4.4 Modeling lipase biosynthesis by ANN

Data of BBD were further modeled using ANN. The resulting theoretical values of the three factors, based on the ANN model, were experimentally validated, which showed better aptness of the ANN.

Artificial intelligence has gained significant prominence in recent scientific research. In this connection, the ANN employs a machine learning paradigm together with a flexible function for the prediction of the input, and output variables [33]. In our research topic, there is no prior research on modeling microbial lipase biosynthesis using ANN. This study represents the first to support this type of modeling. The main benefits of modeling with ANN are its elasticity, ability to predict well with a suitable architecture, and capacity to learn any nonlinear function. Moreover, ANN is a brilliant modeling approach when it is not essential to explain the correlation between the response(s), and the input(s) [12,13,34,35]. So, ANN can create an efficient model from any data type, provided suitable node(s) and hidden layer(s) are used [14].

This study utilizes a multilayer perceptron, which is a fully connected algorithm for machine learning. This unique architecture uses indirect intermediate layers from the independent variables to predict the target response (lipase). Therefore, ANN is a brilliant modeling approach when it is not essential to explain the correlation between the response(s), and the input(s) [12,13,34,35].

### 4.5 Comparison of the models

To compare the performance of the current two models, a linear regression analysis was conducted. The ANN model's predicted points were found to be nearer to the line of ideal prediction than those of the BBD model, indicating that the ANN model has better generalization capability. Another comparison was made by analyzing the predicted versus residual values of the two models. The equal distribution along both 0-axis sides indicated that the variance of lipase production was independent of the lipase biosynthesis route, confirming the adequacy and generalization capacity of both models. The residuals of the ANN model were found to be more ideal than those of the BBD model.

The regression Equation (2) was graphically constructed in terms of the 3D plots. Lipase biosynthesis was drawn against pairwise factors, which varied within the experimental range while keeping the third factor constant at its center level. The 3D plots displayed the optimum level of each variable based on the hump, on which the optimum value for each variable was determined. The elliptical curve nature of the 3D plot for BBD indicates a strong and clear interaction between every pair of the tested factors, suggesting the three factors and their levels were sensibly chosen, and the model fits the design well [10]. On the other hand, no specific prototype could be drawn between every pair of variables using ANN. This is because ANN can discover complicated nonlinear associations between input and output variables, even when there is no apparent relation between them, owing to the presence of intermediate (hidden) layers in ANN that manage exclusive associations between input(s) and output(s), rather than a direct path [36]. Therefore, artificial neural networks (ANN) excel as predictors in cases where the relationships between inputs and outputs are either unnecessary or unidentifiable [12,19,34,35].

However, the differences in statistics between each model were minimal, consequently, both models can be used for modeling fungal lipase production, but ANN surpassed and has a superior generalization capability compared to BBD. The theoretical response of lipase calculated by both models and desirability values (0.9470 (BBD) and 0.9662 (ANN)), indicated the more validity of both models but, ANN surpassed the BBD model.

The models were compared using the desirability function to determine the ideal predicted conditions for achieving the maximum response. The desirability function values range between 0 (representing undesirability) and 1 (representing desirability). Typically, the mathematical determination of the desirability function value occurs before the validation of the modeling process [31,32]. The results of the validation process indicated that the investigational and predicted values of fungal lipase production were in tremendous agreement, suggesting that the desirability function successfully predicted the optimal conditions for achieving maximum biosynthesis of fungal lipase. However, it was observed that the desirability function was more consistent with the ANN model than BBD.

ANN boasts high predictive accuracy due to its superior handling of system nonlinearity compared to models requiring only one-step calculations to reach the target [12,34]. However, ANN also comes with limitations, including extended computational time due to numerous iterations, and the inability to elucidate the significance and impact of each factor in the model. This complexity can hinder the removal or simplification of factors from the model [12,28].

### 4.6 Role of the tested factors

The current study reported a simple medium for lipase production; thus, the only effective parameters were selected for optimization studies.

Cultural humidity can significantly influence lipase production, as the biosynthesis of lipase-producing microorganisms is highly dependent on the availability of water in the culture medium. Cultural humidity refers to the amount of water vapor present in the air surrounding microorganisms. It is an important factor that affects the growth and metabolism of microorganisms, including lipase production. High humidity levels can lead to the accumulation of water droplets on the surface of the culture medium, which can affect the availability of oxygen and nutrients to the microorganisms. This, in turn, can affect the production of lipase by microorganisms. Studies have shown that the optimum humidity level for lipase biosynthesis by microorganisms varies based on the type of microorganism and the culture conditions. For example, some bacteria and fungi produce more lipase under high-humidity conditions, while others produce more lipase under low-humidity conditions [37].

The surfactants can have a significant impact on lipase production. Surfactant is a substance that lessens the surface interfacial tension between different phases such as between oil and water. They are often used in detergents, emulsifiers, foaming agents, and dispersants. They can have an impact on the ability of lipase to access and break down substrates, as well as affect microbial growth and metabolism. Brij 35 is a non-ionic surfactant that is commonly used in microbial cultures. Many investigations have reported the effect of surfactants on lipase production. For example, a study found that the addition of a surfactant to the culture medium significantly increased the production of lipase by *Aspergillus tamarii*, suggesting that the surfactant improved the availability of the substrate for the lipase enzyme [38]. In contrast, another study found that surfactants such as Tween 80 decreased the lipolytic activity of *Staphylococcus aureus* [39], suggesting that some microbes may not have required surfactants to access the substrate, or the surfactants inhibited cell growth or lipase activity.

Inoculum density, or the concentration of microbial cells used to initiate a culture, can significantly affect enzyme biosynthesis. The optimal inoculum density for lipase production depends on the microbial strain, the culture situations, and the type of lipase being produced, in general, low inoculum densities are associated with longer lag phases and slower growth rates, which can delay the onset of lipase production. However, high inoculum densities can lead to overcrowding and nutrient depletion, which can limit enzyme production [37,40]. Overall, lipase production appears to be strain-specific and dependent on the culture conditions.

### 4.7 Fungal biodiesel

The biodiesel production was performed by the action of *A. flavipes* lipase on corn oil. Biodiesel is a renewable fuel produced from various animal fats, vegetable oils, and waste cooking oil. It is an eco-friendly alternative to traditional diesel fuel as it reduces greenhouse gas emissions and dependency on non-renewable fossil fuels [41].

The production of biodiesel involves a process called transesterification, where the triglycerides (oil or fat) react with alcohol, generally ethanol, or methanol, in the occurrence of a catalyst. Lipase catalyzes the transesterification reaction between triglycerides and alcohol, through a process called enzymatic transesterification, to produce fatty acid methyl esters (FAMEs), which are the key constituents of biodiesel. Lipase starts to hydrolyze the ester bond of the triglycerides, leading to the production of free fatty acid and a diacylglycerol molecule. This step is crucial in the production of biodiesel. The next step involves transesterification, in which alcohol reacts to form a monoacylglycerol and FAME, catalyzed by the same lipase enzyme [25,42]. The composition of FAMEs is used as an indicator of the quality of the biodiesel.

The cost of using chemical catalysts in oil feedstock accounts for 75% of total biodiesel synthesis costs [25]. Thus, lipases can be considered an attractive alternative to chemical catalysts for transesterification reactions. Several key roles of microbial lipase in biodiesel production were reported. These advantages include 1) Improved reaction kinetics of the transesterification process. This is because lipases are highly specific and can selectively esterify the hydroxyl groups of glycerol with an alcohol to produce biodiesel. 2) High yield, with low cost, since microbial lipases can produce high yields of biodiesel even at lower temperatures and without the need for extensive purification of the feedstock, thus reducing the overall cost of biodiesel production. 3) Versatility is another advantage where microbial lipases can work with a wide range of feedstocks, including waste cooking oil, animal fats, and vegetable oils, making them a versatile catalyst for biodiesel production. 4) The use of microbial lipases can decrease the amount of waste generated during the biodiesel production process, as it does not require harsh chemical catalysts that can produce toxic by-products. 5) Microbial lipases can be immobilized onto various supports, such as silica or nylon, which allows for their reuse in multiple reaction cycles, reducing the overall cost of biodiesel production.

## 5 Conclusions

A combination pioneer system composed of the ANN-based approach, SSSF, and a new efficient lipolytic fungus was applied for the first time for lipase biosynthesis. First, the lipase production process was fully optimized using the experimental matrix of BBD. The empirical model of BBD was created based on the three-factor matrix (cultural humidity, the surfactant; Brij, and inoculum density). To examine the potential improvement in the modeling process, the obtained data were further modeled using the artificial intelligence approach of ANN. The optimum SSSF condition for lipase biosynthesis varied according to the model used. Experimental validation of the modeling process revealed a high lipase yield of 209.13 ± 3.27, and 218 ± 2.01 U for BBD, and ANN, respectively. Interestingly, the ANN model was more efficient and surpassed the BBD model in the prediction accuracy of the best fermentation conditions that maximize lipase biosynthesis. The ANN optimum conditions were 5.4 ml water/g (cultural humidity), 46.6 μl/g (surfactant; Brij 35), and 10^8^ spore/g inoculum density. Utilizing the ANN prediction model, the synthesized fungal lipase was effectively harnessed for the conversion of corn oil into biodiesel, resulting in a yield of 29.5 mg/l. This innovative approach offers a promising solution for generating sustainable energy and stands as a significant advancement in the field of biofuel research. Despite the use of lipase in biodiesel production having numerous merits over conventional chemical catalysts, the economic viability of large-scale production, as compared to the standard technique, remains a challenge that needs to be addressed and clarified before it can be considered a profitable outcome. Extended work is encouraged to test the ability of the resulting lipase to produce biodiesel from several kinds of oils. Another, the purification of the enzyme may improve the bioconversion efficacy of the enzyme.

## Author contributions

Conceived and designed the experiments; Mohammad El-Metwally, Gamal Abdel-Fattah, and WesamEldin Saber. Performed the experiments, Mohammad El-Metwally, Dina EL-Khatieb, Yosra Helmy, and WesamEldin Saber. Analyzed and interpreted the data; Mohammad El-Metwally, Fatimah Al-Otibi, Dina EL-Khatieb, and Youssef Mohammed. Contributed reagents, materials, analysis tools, or data; Fatimah Al-Otibi, Dina EL-Khatieb, and Youssef Mohammed. Drafting the article or critically revising its important intellectual content; all authors. Wrote the paper; all authors. Final approval of the version submitted; all authors.

## Data availability statement

No data was used for the research described in the article.

## Declaration of competing interest

We are enclosing herewith an original research article entitled “**Application of artificial neural networks for enhancing *Aspergillus flavipes* lipase synthesis for green biodiesel production”** for consideration by ***Heliyon***. We confirm that this work is original and neither the manuscript nor any part of its content is currently under consideration or published in another journal. We declare that there is no conflict of interest. We have no conflict of interest to disclose. All authors have participated and approved the manuscript and agree with its submission to ***Heliyon***. Thanks for considering the manuscript.
